# Kinetics of gas production in the presence of *Fusarium* mycotoxins in rumen fluid of lactating dairy cows

**DOI:** 10.3168/jdsc.2021-0100

**Published:** 2021-05-21

**Authors:** A. Gallo, F. Ghilardelli, B. Doupovec, J. Faas, D. Schatzmayr, F. Masoero

**Affiliations:** 1 Department of Animal Science, Food and Nutrition (DIANA), Faculty of Agricultural, Food and Environmental Sciences, Università Cattolica del Sacro Cuore, 29122 Piacenza, Italy; 2 Biomin Research Center, Technopark 1, 3430 Tulln, Austria

## Abstract

Little is known about the effects of *Fusarium* mycotoxins on the fermentation potential of rumen fluid sampled from lactating dairy cows ingesting diets contaminated at regular levels of these mycotoxins (i.e., contamination levels that can normally be found on dairy farms). In the current experiment, rumen donor animals received diets contaminated with both deoxynivalenol (DON) and fumonisins (FB) with or without a mycotoxin-deactivating product. The rumen fluid donor animals were 12 lactating Holstein dairy cows that received one of 3 experimental diets in agreement with a 3 × 3 Latin square design (3 periods and 3 treatments). The 3 diets were as follows: (1) a TMR contaminated with a regular level of *Fusarium* mycotoxins [340.5 ± 161.0 µg of DON/kg of dry matter (DM) and 127.9 ± 43.9 µg of FB/kg of DM; control diet, CTR], (2) a TMR contaminated with *Fusarium* mycotoxins at levels higher than CTR but below US and European Union guidelines (733.0 ± 213.6 µg of DON/kg of DM and 994.4 ± 323.2 µg of FB/kg of DM; MTX), and (3) the MTX diet (897.3 ± 230.4 µg of DON/kg of DM and 1,247.1 ± 370.2 µg of FB/kg of DM) supplemented with a mycotoxin-deactivator product (Mycofix, Biomin Holding GmbH; 35 g/animal per day; MDP). Each experimental period lasted 21 d, and rumen fluid was individually sampled from all cows on the last day of each intoxication period. Then, the 4 rumen fluids sampled from cows receiving the same experimental diets were pooled into a single rumen inoculum, which was used in the in vitro gas production test. For the gas production test, 3 different rumen inocula (i.e., CTR, MTX, and MDP) were buffered (buffer:rumen ratio of 2:1, vol/vol) and then used in 3 fermentation runs to evaluate gas production dynamics in the presence of 8 feeds (i.e., corn meal, barley meal, corn silage, sorghum silage, alfalfa hay, ryegrass hay, dry brewers barley grains, and dried distillers grains with solubles). The kinetic parameters of gas production and volatile fatty acid concentrations were evaluated at the end of fermentation. The block run (i.e., fermentation day) effect influenced all of the fermentative and kinetic parameters. Greater final volumes or rates of gas production over time were observed for MDP compared with MTX rumen inocula (i.e., 172.6 vs. 147.8 mL/g of organic matter or 0.078 vs. 0.063 h^−1^, respectively). However, the increase in rate of gas production was not consistent among tested feeds, meaning that a treatment by feed interaction was observed. Volatile fatty acid concentrations were not different among treatments, except for a slight increase of acetic acid in CTR compared with MTX (i.e., 71.0 vs. 67.9 mmol/L). This study showed that *Fusarium*-produced mycotoxins negatively affected the kinetics of gas production in feeds, whereas the presence of the mycotoxin-deactivator product in the diets of donor animals resulted in an increase in rumen fermentation potential, thus safeguarding the rumen environment.

## Highlights

• Toxins produced by *Fusarium* can be commonly detected in ruminant diets.
• Deoxynivalenol and fumonisins in the diet interfere with rumen microbiota.
• The presence of a mycotoxin-deactivating product counteracted negative effects.

## Graphical Abstract

**Figure:**
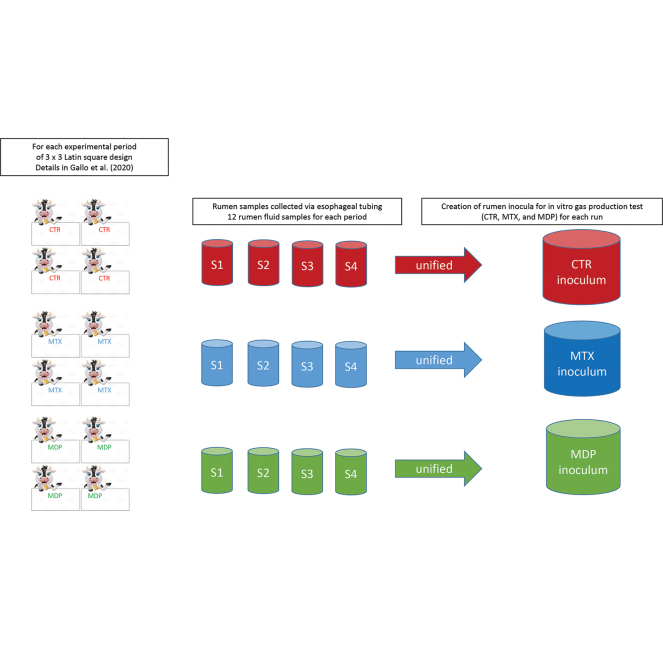
**Summary:** Little is known about the effects of *Fusarium* mycotoxins, such as deoxynivalenol and fumonisins, on rumen fermentation potential. The aim of this study was to understand how these mycotoxins affect the fermentation kinetics and volatile fatty acid concentrations of different feeds incubated in rumen fluids from lactating dairy cows ingesting contaminated diets (CTR, MTX, and MDP) at regular contamination levels (i.e., contamination levels that can be commonly detected in dairy cow diets). The presence of *Fusarium*-produced mycotoxins reduced rumen fluid fermentation potential and slowed the degradation dynamics of incubated feeds. The presence of a mycotoxin-deactivating product enabled the protection of the rumen environment.

Mycotoxins are secondary metabolites of filamentous fungi belonging to *Aspergillus*, *Fusarium*, *Penicillium*, and other fungal genera. When ingested by humans and other vertebrates, they may have carcinogenic, mutagenic, teratogenic, or immunosuppressive effects depending on the characteristics of the mycotoxin structure (Jouany and Diaz, 2005). Among mycotoxins, those produced by *Fusarium* spp. are usually detected in both concentrate and forage (Cheli et al., 2013; Gallo et al., 2015) because mycotoxigenic *Fusarium* molds are widespread and able to contaminate field crops in temperate and warm climate zones (EFSA, 2013). These mycotoxins are characterized by having either antibacterial or antifungal activities (Venkatesh and Keller, 2019). In particular, *Fusarium* strains produce a high number of harmful mycotoxins, such as zearalenone, deoxynivalenol (**DON**), and fumonisins (**FB**). Compared with most animals, ruminants are considered to be less sensitive to *Fusarium*-produced mycotoxins because rumen microorganisms have the potential to detoxify these mycotoxins (Fink-Gremmels and Diaz, 2005; Fink-Gremmels, 2008). On the other hand, functionally important and abundant rumen microorganisms, such as *Ruminococcus albus* or the methanogenic archaeon *Methanobrevibacter* spp., can be inhibited by *Fusarium*-produced toxins (May et al., 2000). Growth inhibition by DON or FB has also been reported for other bacteria strains, such as members of the genera *Lactobacillus*, *Bacillus*, and *Streptococcus* (Ali-Vehmas et al., 1998). As previously discussed (Strobel et al., 2008), the inhibiting effects of mycotoxins on bacteria have been tested under pure culture laboratory conditions (i.e., no feed matrix involved in the study), but it remains unclear how *Fusarium*-contaminated feeds would affect the rumen microbial community as well as its ability to ferment OM or specific nutrients. Thus, the objective of this study was to determine the effect of *Fusarium*-produced mycotoxins, mainly DON and FB, on the kinetics of gas production in the presence of different feeds by using rumen inocula sampled from donor lactating dairy cows receiving contaminated diets with or without a mycotoxin-deactivating product.

The in vivo study was authorized by the Italian Health regulations that pertain to the accommodation and care of animals used for experimental and other scientific purposes (authorization no. 232/2017-PR issued on Mar. 17, 2017). The details regarding experimental conditions and trial organization management were previously detailed by Gallo et al. (2020). In particular, 12 multiparous mid-lactation Holstein cows (parity 2.92 ± 1.08; 114 ± 16 DIM; milk yield of 42.2 ± 5.2 kg at study onset) were used in this experiment, which was carried out at the CERZOO research and experimental center (San Bonico, Piacenza, Italy). Cows were kept in a common pen, had free access to water, and were individually fed assigned diets formulated according to the nutrient requirements of dairy cattle (NRC, 2001) using the Calan Broadbent feeding system (American Calan Inc.).

A TMR was used in the in vivo study (Gallo et al., 2020), and the components were mixed in an experimental mixer wagon (Storti model Labrador 70 horizontal mixer, capacity of 8 m^3^; Feraboli Zootech srl) in the following order: corn silage, hays, soybean meal (44%), dehulled sunflower meal (34%), and water. The different treatments were created by manually mixing and adding one of the 2 corn meals (i.e., normal or supplemented with a mycotoxin-contaminated culture material) specific to the experimental diets. Then, diets were individually fed to animals in specific feeding stations. The corn supplemented with a contaminated culture material contained FB from *Fusarium verticillioides* (Desjardins and Plattner, 2000) that was grown on rice, homogenized, and dried or DON from *Fusarium graminearum* (Altpeter and Posselt, 1994) that was grown on maize, homogenized, and dried. The whole TMR, with different corn meals depending on the specific treatment assigned to cows in a specific period, was provided individually based on measurement of DMI on the previous day. The whole study lasted 112 d and consisted of a group formation and adaptation period (21 d) and three 21-d experimental periods (in which each cow was assigned to a specific treatment) that were separated by two 14-d clearance periods. During the adaptation and clearance periods, all animals received the same TMR diet, including normal corn meals. The animals alternatively received one of 3 experimental diets in agreement with a 3 × 3 Latin square design (3 periods and 3 treatments). Three cows were randomly allocated to one of 4 Latin squares (total number of cows = 12), and dietary treatments consisted of the following: (1) a TMR contaminated with a regular level (i.e., contamination level that can be commonly detected in dairy cow diets) of *Fusarium* mycotoxins [340.5 ± 161.0 µg of DON/kg of DM and 127.9 ± 43.9 µg of FB/kg of DM; control diet, **CTR**], (2) a TMR contaminated with *Fusarium* mycotoxins at levels higher than CTR but below US and European Union guidelines (733.0 ± 213.6 µg of DON/kg of DM and 994.4 ± 323.2 µg of FB/kg of DM; **MTX**), and (3) the MTX diet (897.3 ± 230.4 µg of DON/kg of DM and 1,247.1 ± 370.2 µg of FB/kg of DM) supplemented with a mycotoxin-deactivator product (Mycofix, Biomin Holding GmbH; 35 g/animal per day; **MDP**). Only numerical differences in DON and FB contamination levels between the MTX and MDP diets were measured, probably due to high variability related to mycotoxin quantification in a complex matrix such as TMR (Steiner et al., 2020).

A ruminal fluid sample (200 mL) was collected from each animal 5 to 6 h after the morning feeding on the last day of each experimental period (d 21). This sampling interval was characterized by great availability of fermentable substrate, which leads to an increase in fermentation rate or VFA production and concentration as well as a low pH of rumen fluid (de Assis Lage et al., 2020). These samples were collected using a stomach tube that was connected to a manual vacuum pump (Gallo et al., 2020). The rumen fluids sampled from the animals consuming the same diets (i.e., 4 each for CTR, MTX, and MDP) were pooled, filtered through 3 layers of cheesecloth, and used as rumen inocula in the gas production test within 30 min from sampling.

In the gas production test, 8 feeds were separately incubated. The evaluated feeds were samples of corn meal, barley meal, corn silage, sorghum silage, alfalfa hay, ryegrass hay, dry brewers barley grains, and dried distillers grains with solubles. To measure rumen fermentability (gas production), each tested feed was incubated in the 3 diluted rumen fluids (buffer:rumen ratio of 2:1, vol/vol) obtained from cows fed the CTR, MTX, and MDP diets (Menke and Steingass, 1988). Briefly, about 220 mg of each feed sample was weighed in 100-mL bottles. Then, 30 mL of rumen inoculum was dispensed into the bottles containing the samples for a corresponding headspace volume of 70 mL. This procedure was conducted by flushing the bottle headspace with CO_2_. The bottles were hermetically closed with rubber tops and placed in a water batch at 39°C, and gas production was measured after 1, 2, 4, 7, 18, 28, 42, and 76 h of incubation. The bottles were manually agitated at each measurement (Masoero et al., 2010).

The kinetics of gas production were computed using a single-pool exponential model (Wang et al., 2013), in agreement with the following differential equation:

[1] V(*t*) = V*f* × [1 − *e*^−kd × (*t* − *lag*)^],

where V(*t*) is the volume of gas accumulated (mL/g of OM) at time *t*; Vf is the final gas volume (mL/g of OM); **kd** is the kinetic constant (h^−1^); *t* is time (h); and *lag* is the lag time (h). Samples were incubated in triplicate in 3 separate runs, corresponding to d 21 of each in vivo experimental period. Samples within the run were considered analytical repetitions, and samples between runs were considered experimental replicates.

The kinetic parameters of gas production or VFA concentrations measured at the end of the gas production test were evaluated using a completely randomized block design with a factorial arrangement of main effects using the GLM procedure of SAS (SAS Institute, 2004) according with the following model:

*Y_ijkl_* = µ + *R_i_* + *T_j_* + *M_k_* + (*T* × *M*)*_jk_* + *e_ijkl_*,

where *Y_ijkl_* is the response variable (i.e., Vf, kd, lag, or VFA concentrations); µ is the overall mean; *R_i_* is the fixed effect of fermentation run (*i* = 3); *T_j_* is the fixed effect of treatment (*j* = 3: CTR, MTX, and MDP); *M_k_* is the fixed effect of the tested feeds (*k* = 8); (*T* × *M*)*_jk_* is the first-order interaction; and *e_ijkl_* is the random residual error. Differences were presented as the average of the main tested effects, except when first-order interaction was significant at the declared level, and they were compared post hoc using Tukey's honestly significant difference test. If the interaction was significant at *P* < 0.05, a graphical representation of mean differences for the specific parameter of interest was reported.

The characteristics of rumen fluids from donor animals used in the in vivo trial were previously reported in Table 6 of Gallo et al. (2020). The average pH was 6.21, and concentrations of acetic, propionic, and butyric acids were 68.5, 26.3, and 14.9 mmol/L, respectively. No differences were observed on these data among periods or treatments, except for isovaleric acid, which differed in both quantity and proportion among experimental periods. Consequently, the diluted rumen inocula used in the gas production trial could be considered to be similar. Table 1 shows the kinetic parameters of gas production for different feeds as well as concentrations of VFA after 76 h of incubation. The block run effect influenced (*P* < 0.05) all tested parameters. This was probably due to changes in animal feeding behaviors as well as in cow performance during the trial. In particular, the DMI of rumen donor animals decreased from 27.51 to 23.68 kg/cow per day and milk yield decreased from 40.69 to 33.82 kg/cow per day (Gallo et al., 2020) when passing from first to last experimental periods. When DMI decreases, a lower passage rate of nutrients through the gastrointestinal tract and a consequent increase in mean retention time of feeds in the rumen–reticulum compartment is expected, thus modifying the dynamics of nutrient fermentation in the rumen–reticulum (Seo et al., 2006; Muñoz-Tamayo et al., 2016; Sauvant and Nozière, 2016).

**Table 1:** Effect of experimental treatments and inoculated feeds on kinetic gas production parameters and VFA

Item^1^	Treatment^2^ (T)			Feed (F)								SEM	*P*-value			
	CTR	MTX	MDP	Co-product		Hay		Meal		Silage			Run	T	F	T × F
				Brewers	DDGS^3^	Ryegrass	Alfalfa	Corn	Barley	Corn	Sorghum					
Vf (mL)	161.7^ab^	147.8^b^	172.6^a^	190.3^ab^	147.8^c^	133.4^c^	120.4^c^	222.2^a^	196.2^ab^	155.6^bc^	124.7^c^	16.16	<0.05	<0.05	<0.05	0.345
kd (h^−1^)	0.067	0.063	0.078	0.104	0.059	0.057	0.059	0.083	0.092	0.061	0.039	0.0070	<0.05	<0.05	<0.05	<0.05
Lag (h)	0.6	0.7	0.4	0.4^ab^	0.2^b^	0.2^b^	0.4^ab^	1.0^a^	0.8^ab^	0.7^ab^	0.7^ab^	0.28	<0.05	0.221	<0.05	0.926
Acetic acid (mmol/L)	71.0^a^	67.9^b^	68.6^ab^	72.4abc	69.2bcd	67.1cde	62.6^ed^	78.5^a^	74.3^ab^	67.1cde	61.3^e^	2.55	<0.05	<0.05	<0.05	0.466
Propionic acid (mmol/L)	23.9	22.9	23.2	26.5^a^	25.0^a^	21.9^b^	20.6^b^	26.2^a^	24.8^a^	21.6^b^	19.8^b^	0.94	<0.05	0.083	<0.05	0.134
Butyric acid (mmol/L)	19.0	18.1	18.1	20.8^ab^	19.3abc	15.9^cd^	16.2^cd^	21.1^a^	20.6^ab^	17.7bcd	15.8^d^	1.32	<0.05	0.256	<0.05	0.762
C2/C3	2.99	2.99	2.99	2.75^b^	2.77^b^	3.08^a^	3.04^a^	3.00^ab^	3.01^ab^	3.13^a^	3.11^a^	0.102	<0.05	0.989	<0.05	0.991
(C2 + C4)/C3	3.78	3.79	3.77	3.53^b^	3.54^b^	3.81^a^	3.82^a^	3.82^a^	3.84^a^	3.96^a^	3.91^a^	0.077	<0.05	0.918	<0.05	0.863

a–e Within each main factor (i.e., treatment or feeds), means with different superscripts differ (*P* < 0.05).

1 Vf = final gas volume; kd = kinetic constant; lag = lag time.

2 CTR = TMR contaminated with a regular level of *Fusarium* mycotoxins (340.5 ± 161.0 μg of DON/kg of DM and 127.9 ± 43.9 μg of FB/kg of DM; control diet); MTX = TMR contaminated with *Fusarium* mycotoxins at levels higher than CTR but below US and European Union guidelines (733.0 ± 213.6 μg of DON/kg of DM and 994.4 ± 323.2 μg of FB/kg of DM); MDP = MTX diet (897.3 ± 230.4 μg of DON/kg of DM and 1,247.1 ± 370.2 μg of FB/kg of DM) supplemented with a mycotoxin-deactivator product (Mycofix, Biomin Holding GmbH; 35 g/animal per day).

3 Dried distillers grains with solubles.

The effect of *Fusarium*-produced toxins on rumen fermentation parameters and nutrient rumen digestibility is still controversial. In our previous review, we summarized results from several rumen-based in vitro experiments in which the effect of mycotoxins on rumen microbiota was tested (Gallo et al., 2015). The trials strongly differed with regards to experimental conditions and doses at which the specific mycotoxin was tested as well as the source of mycotoxin (i.e., from pure chemical standards, spiked materials, or natural contaminated feeds). In particular, Boguhn et al. (2010) studied the effects of both concentrate proportion and DON-contaminated triticale in the diet on nutrient degradation, microbial protein synthesis, and the microbial community using a rumen-simulation technique. As a result, the inclusion of contaminated triticale did not influence the fermentation of OM or the synthesis and composition of microbial protein. On the contrary, the fermentation of detergent fiber fractions was lower in diets containing contaminated compared with uncontaminated triticale. None of the microbial groups were affected by the presence of DON during the in vitro test, except for the *Clostridia* group; this supported the observed reduction in cellulolytic activity during 48 h of fermentation. In Hildebrand et al. (2012), the supplementation of FB in rumen inoculum had only marginal effects on the characteristics of gas production, even if a significant but small effect on the shapes of gas production curves was observed in the presence of these mycotoxins. Jeong et al. (2010) observed that DON negatively affected certain aspects of rumen fermentative capacity, such as ammonia-N and total gas production. Further, acetate and propionate productions were reduced. May et al. (2000) did not report a synergistic inhibitory effect of DON and fusaric acid, even if the latter inhibited the growth of *Ruminococcus albus* and *Methanobrevibacter ruminantium*. Other studies (Auerbach et al., 1998; Gurung et al., 1999; Morgavi et al., 2013) reported that *Fusarium*-produced toxins had no effect on in vitro DM degradability. In all previously cited studies in this paragraph, the tested mycotoxin was directly added to buffered rumen fluid at the beginning of the respective in vitro test. In the current study, both mycotoxins as well as a mycotoxin-deactivator product were mixed into the diets that the rumen fluid donor animals ingested. To the best of our knowledge, this is the only study in which this experimental condition has been used.

The use of a mycotoxin-deactivator product in the diet of donor animals changed the kinetics profile of the tested feeds. The mycotoxin-deactivator product has been described in detail in Gallo et al. (2020). It contains a clay mineral-based adsorbent capable of binding aflatoxins, the bacterial strain Biomin BBSH797 capable of deactivating trichothecenes, and a biological constituent and a purified enzyme (fumonisins esterase, FUMzyme, Biomin) to counteract zearalenone (ZEN) and FB, respectively. Furthermore, a blend of extracts from plants and algae is included in the product. The final gas volume increased (*P* < 0.05) by approximately 6 and 14% in MDP compared with CTR and MTX, respectively. A greater kd value was observed for MDP than for CTR or MTX (i.e., 0.078 vs. 0.067 and 0.063 h^−1^, respectively; *P* < 0.05), even if not consistently among feeds. As shown in Figure 1, the majority of feeds were more slowly fermented in MTX compared with the other 2 rumen inocula (CTR and MDP), even if barley increased kd in this treatment. With some other feeds (i.e., alfalfa hay and grass hay), the kd remained almost unchanged among treatments. More than a change in bacterial community when incubating samples, this could be related to specific nutritional characteristics of tested feed as well as specific adopted experimental conditions. The VFA concentration did not change among treatments, except for acetic acid, which was slightly higher in CTR than in MTX (i.e., 71.0 vs. 67.9 mmol/L). Although the differences were statistically significant, the biological relevance of such a numerically low difference is negligible.

**Figure 1:**
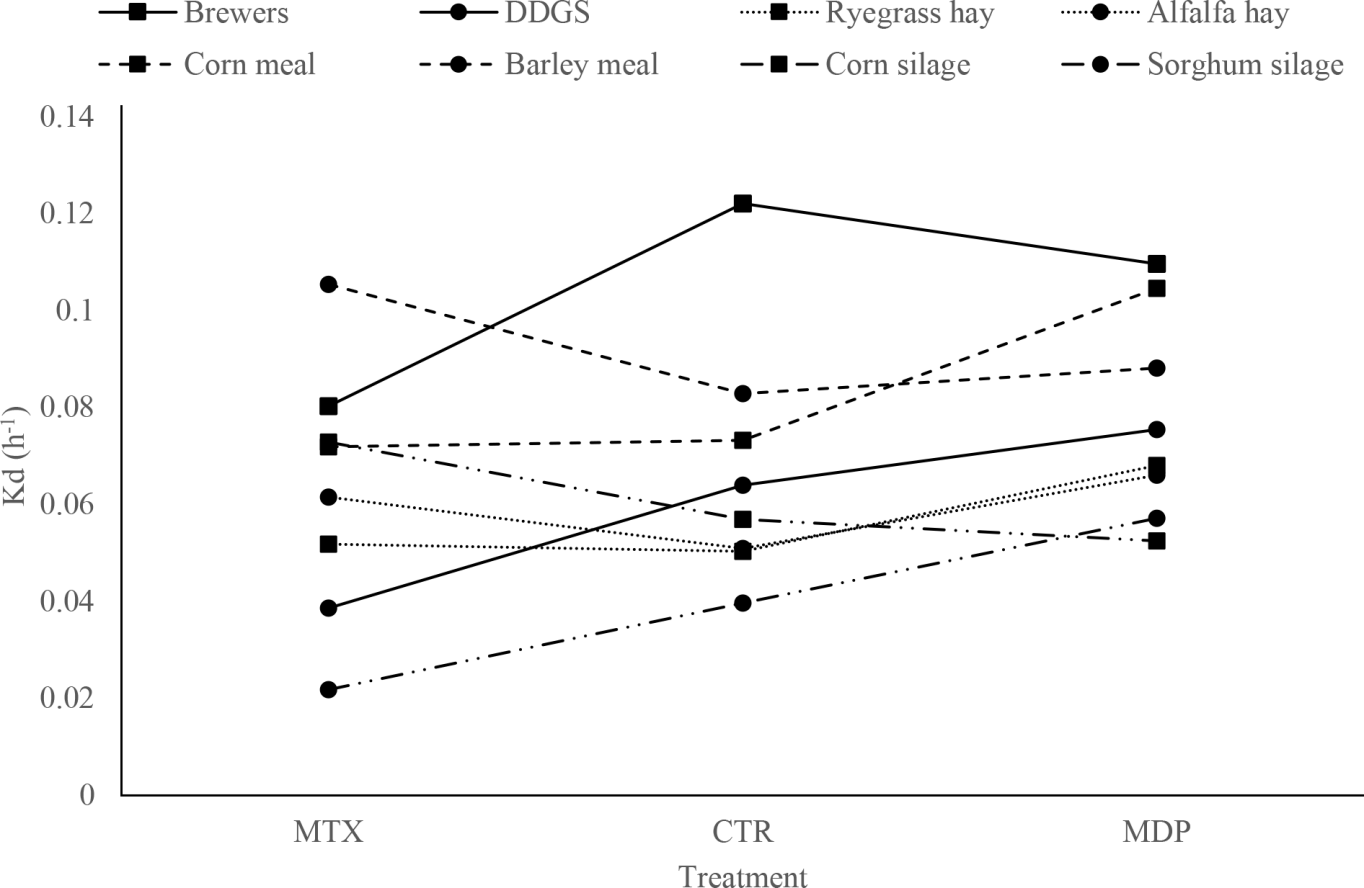
Interaction between treatments and feeds on rate of gas production (kd; h^−1^). CTR = TMR contaminated with a regular level of *Fusarium* mycotoxins [340.5 ± 161.0 µg of DON/kg of DM and 127.9 ± 43.9 µg of FB/kg of DM; control diet]; MTX = TMR contaminated with *Fusarium* mycotoxins at levels higher than CTR but below US and European Union guidelines (733.0 ± 213.6 µg of DON/kg of DM and 994.4 ± 323.2 µg of FB/kg of DM); MDP = the MTX diet (897.3 ± 230.4 µg of DON/kg of DM and 1,247.1 ± 370.2 µg of FB/kg of DM) supplemented with a mycotoxin-deactivator product (Mycofix, Biomin Holding GmbH; 35 g/animal per day). DDGS = dried distillers grains with solubles.

In conclusion, the presence of *Fusarium*-produced mycotoxins, mainly DON and FB, in the diet of rumen fluid donor animals affected the kinetics of gas production of different feeds, thus corroborating the hypothesis that these mycotoxins have a direct effect on rumen microbiota. The presence of a mycotoxin-deactivator product demonstrated the ability to safeguard the rumen environment and increase fermentation dynamics of feeds.
